# Editorial: Pharmaceutical materials for tumor imaging and therapy

**DOI:** 10.3389/fbioe.2022.1099762

**Published:** 2022-12-08

**Authors:** Wenliang Li, Junqing Wang, Jianxun Ding, Na Kong, Leijiao Li, Yu Sun, Bo Zhou

**Affiliations:** ^1^ Key Laboratory of Jinlin Province for Zoonosis Prevention and Control, Military Veterinary Institute, AMMS, Changchun, China; ^2^ Jilin Collaborative Innovation Center for Antibody Engineering, Jilin Medical University, Jilin, China; ^3^ School of Pharmaceuticals Sciences (Shenzhen), Sun Yat-sen University, Guangzhou, China; ^4^ Key Laboratory of Polymer Ecomaterials, Changchun Institute of Applied Chemistry, Chinese Academy of Sciences, Changchun, China; ^5^ Center for Nanomedicine and Department of Anesthesiology, Brigham and Women’s Hospital, Harvard Medical School, Boston, MA, United States; ^6^ School of Chemistry and Environmental Engineering, Changchun University of Science and Technology, Changchun, China

**Keywords:** pharmaceutical material, imaging-guided therapy, photo-sensitive agent-mediated therapy, gene therapy, pharmacokinetics

## 1 Introduction

Biomedicine is undergoing a complete transformation, brought about by the development of nanotechnology. Cancer is a major public health problem worldwide. Therefore, a wide variety of pharmaceutical nanomaterials exhibiting antitumor activities have been designed and prepared for the improvement of human health. In this Research Topic “Pharmaceutical Materials for Tumor Imaging and Therapy”, we focused on diagnostic imaging, available therapies, pharmaco-mechanisms, and pharmacokinetics specific to antitumor pharmaceutical materials. Nine articles are presented and are divided into five themes: 1) imaging-guided therapy, 2) photo-sensitive agent-mediated therapy, 3) gene therapy, 4) pharmacokinetics, and 5) research progress in pharmaceutical materials.

## 2 Imaging-guided therapy

One article has been published related to imaging-guided therapy. Xin et al. designed and prepared an iodine (I)-rich amphiphilic copolymer, poly(ethylene glycol)-poly(2‐hydroxyethyl methacrylate)-I (PEG-PHEMA-I, INA), for computed tomography (CT) imaging–guided photo-dynamic immunotherapy of breast cancer. INA, as a CT contrast agent, successfully illuminated the tumors in CT imaging. Under the guidance of CT imaging, photo-dynamic therapy (PDT) triggered by INA induced immunogenic cell death (ICD) to trigger the release of immune-associated cytokines. Good anticancer efficacy both *in vitro* and *in vivo* were obtained.

## 3 Photo-sensitive agent-mediated therapy

With the rapid development of nanotechnology, various photo-sensitive nano-agents have been added to pharmaceutical nanomaterials. Photo-sensitive nano-agents cover a wide range of materials, such as photo-responsive nanoparticles and photo-sensitizers for photo-thermal or photo-dynamic therapy (PTT/PDT). For example, a series of thermo- and light-responsive copolymers consisting of poly(*N*-isopropylacrylamide) (PNIPAM) and 6-[4-(4-methoxy phenyl azo)-phenoxyl-hexyl methacrylate) (AzoMA); PNIPAM-*b*-PAzoMA, were synthesized by Cui et al. *via* reversible addition-fragmentation chain transfer (RAFT) radical polymerization. PNIPAM-*b*-PAzoMA was used as a carrier for the delivery of ferroferric oxide (Fe_3_O_4_) nanoparticle. The photo-sensitive agent showed no significant cytotoxicity and good stability in physiological environments, demonstrating its potential for cancer therapy.

Wang et al. reported a charge-reversal nanoplatform (chlorin e6-poly(lactic-co-glycolic acid)@polydopamine-poly(allylamine hydrochloride)-dimethyl maleic acid nanoparticle (Ce6-PLGA@PDA-PAH-DMMA NP)), including PDA and Ce6 for enhancing synergistic PTT/PDT. The PAH-DMMA charge-reversal layer enabled Ce6-PLGA@PDA-PAH-DMMA NP to circulate for a long time in the blood at normal physiological condition and to successfully realize charge reversal in a weakly acidic tumor microenvironment, thereby improving cell uptake. This strategy provided a promising approach for the synergistic PTT/PDT for breast cancer.

## 4 Gene therapy

Delving further into the study of cancer pathogenicity mechanisms, researchers have realized that tumors are a type of gene disease. In the past two decades, approximately 1,000 clinical cancer trials based on gene drugs have been conducted. Yang et al. investigated the effects of sulfured polysaccharide from Undaria pinnatifida (SPUP) on the biological behaviors of ovarian cancer cells. The authors found that SPUP inhibited the proliferation, migration, and invasion of ovarian cancer cells, and induced their apoptosis by inhibiting the activation of Hedgehog signaling pathway at the protein level. In light of this discovery, natural products, particularly SPUP may be utilized as gene therapeutic agents for cancer therapy.

## 5 Pharmacokinetics

Doxorubicin (DOX), a potent anthracycline cytotoxic drug, has been routinely used as a frontline chemotherapeutic agent for the treatment of various cancers. The article by Xu et al. reported the molecular dynamic behaviors of free DOX and DOX-conjugated lipid prodrug molecule using molecular dynamics simulations. The authors concluded that free DOX loaded in a nanodisc system experienced rapid dissociation due to the unfavorable orientation of DOX attached to the lipid surface. The authors also investigated the conformational variation of nanodisc components, as well as intermolecular interactions, solvent accessible surface areas, and the flexibility of the individual membrane scaffold protein 1 residue.

A lipiodol nanoformulation, to overcome the drawbacks of interventional embolization chemotherapy, was introduced by Peng et al. The study demonstrated that superstable, homogeneous, and intermixed formulation technology allowed the clever combination of lipiodol and hydrophilic chemotherapeutic drugs to prepare an effective and superstable homogeneous lipiodol and DOX, exhibiting improved clinical transformation and application value.

## 6 Research progress in pharmaceutical materials

This sub-theme covers three reviews related to recent research progress in pharmaceutical materials. For example, Xiao et al. surveyed recent advances in research on polymeric nanoparticles used for controlled cancer drug delivery. This research reviewed the current state of cancer drug loading systems by presenting a series of published articles that highlighted the novelty and functions of a variety of different architectures, including micelles, liposomes, dendrimers, polymersomes, hydrogels, and metal-organic frameworks. This article may contribute to the development of useful polymeric nanoparticles to achieve different therapeutic purposes. Moreover, another article summarized the recent research on lipid nanoparticle (LNP) vehicles, utilized as powerful mRNA delivery tools for mRNA cancer therapy. The formulation components of mRNA-LNPs were discussed, and future challenges and directions were also highlighted. Furthermore, Wang et al. performed a systematic review of inorganic nanomaterials for the prevention and treatment of bacterial infection. Several classical, metal-based, metal-like, and carbon-based nanomaterials, used as PTT agents, were reviewed, and their advantages were discussed and summarized. These discussions may provide valuable suggestions for future research on near-infrared (NIR) photo-thermal conversion inorganic nanomaterials.

## 7 Conclusion

In summary, this Research Topic on “*Pharmaceutical Materials for Tumor Imaging and Therapy*” presents articles on different types of pharmaceutical materials for diagnosis and treatment of cancers. These articles describe advances in tumor imaging and therapy, highlighting exciting and transformative accomplishments.

